# A transgenic embryonic sexing system for the Australian sheep blow fly *Lucilia cuprina*

**DOI:** 10.1038/srep16090

**Published:** 2015-11-05

**Authors:** Ying Yan, Maxwell J. Scott

**Affiliations:** 1 Department of Entomology, North Carolina State University, Campus Box 7613, Raleigh, NC, 27695-7613.

## Abstract

Genetic approaches, including the sterile insect technique (SIT), have previously been considered for control of the Australian sheep blow fly *Lucilia cuprina*, a major pest of sheep. In an SIT program, females consume 50% of the diet but are ineffective as control agents and compete with females in the field for mating with sterile males, thereby decreasing the efficiency of the program. Consequently, transgenic sexing strains of *L. cuprina* were developed that produce 100% males when raised on diet that lacks tetracycline. However, as females die mostly at the pupal stage, rearing costs would not be significantly reduced. Here we report the development of transgenic embryonic sexing strains of *L. cuprina*. In these strains, the *Lsbnk* cellularization gene promoter drives high levels of expression of the tetracycline transactivator (tTA) in the early embryo. In the absence of tetracycline, tTA activates expression of the *Lshid* proapoptotic gene, leading to death of the embryo. Sex-specific RNA splicing of *Lshid* transcripts ensures that only female embryos die. Embryonic sexing strains were also made by combining the *Lsbnk*-tTA and tetO-*Lshid* components into a single gene construct, which will facilitate transfer of the technology to other major calliphorid livestock pests.

*Lucilia cuprina* is a serious economic pest for the sheep industry in Australia and New Zealand^1^. Larvae that develop from eggs laid from *L. cuprina* females on sheep cause a cutaneous myiasis (maggot-infested wounds), which can lead to reduced wool production and death of the animal if not treated. The success of the New World screwworm (*Cochliomyia hominivorax*) sterile insect technique (SIT) program^2^,^3^ encouraged the development of genetic approaches for control of *L. cuprina*^4^. *C. hominivorax*, a blow fly that is related to *L. cuprina*^5^, also causes myiasis, which can lead to lethality if untreated^6^. However, *C. hominivorax* is an obligate parasite and is a devastating pest of warm-blooded mammals including several livestock species. Over an approximately 50-year period, the SIT was used to eradicate *C. hominivorax* from the U.S.A, Mexico, and Central America^2^,^3^. SIT involved mass rearing of *C. hominivorax*, sterilization of males and females by exposure to high doses of gamma irradiation and controlled release of the sterile males and females over the targeted area. As the sterile males were in a large excess (at least 9:1) over fertile males in the field, fertile females in the release area were more likely to mate with the sterile males^7^. Currently, sterilized *C. hominivorax* are released along the Panama-Colombian border to prevent re-introduction of the pest from South America.

For genetic control of *L. cuprina* to be cost-effective in Australia, strains were developed that were predicted to be more effective at lower release ratios than a bisexual sterile release. The “field female killing (FFK)” strains carried recessive eye color mutations and Y:autosome translocations that essentially made only the females blind in the field^4^. A field trial on Flinders island in 1985–1986 was successful in significantly reducing the population of *L. cuprina*^4^. However, due to problems in mass-rearing the strain (breakdown of Y:autosome translocations, semi-sterility, fungal infection) and declining wool price, the trial was never extended to mainland Australia. More recently, Mahon has suggested that SIT be reconsidered for control of *L. cuprina*, with an initial emphasis on the island state of Tasmania^8^. An alternative approach for improving the efficiency of SIT is to employ a so-called “genetic-sexing” strain (GSS) that would facilitate mass separation of males and females so that only sterile males would be released in the field. GSS are widely used for Mediterranean fruit fly (*Ceratitis capitata*) SIT programs^9^. It has been shown that sterile male-only *C. capitata* releases are 3–5 times more effective in the field than bisexual releases^10^,^11^. GSSs employ recessive pupal color or temperature sensitive lethal (*tsl*) mutations and Y: autosome translocations where a functional copy of the gene (e.g. *tsl*^+^) is linked to the Y centromere. A disadvantage of GSS strains is that they are prone to breakdown due to rare recombination events in males^9^. Further, assembling a GSS requires isolation of suitable recessive mutations and chromosome rearrangements, which are not portable to other related pest species.

An alternative to GSS is to employ transgenic technology to make conditional male-only strains^12^,^13^. Assembling transgenic sexing strains (TSS), requires a conditional gene expression system for controlling expression of a gene that is lethal to the insect. Further, expression of the lethal gene must be sex-specific such that only females die. In the initial system we developed, the tetracycline transactivator (tTA) was employed to activate expression of the *hid* proapoptotic gene in *Drosophila melanogaster*^14^. *hid* expression was driven by an enhancer-promoter that comprised seven copies of the tTA binding site (tetO) and the *hsp70* core promoter. tTA expression was driven by the female-specific transcription enhancer from the *yolk protein* 1 gene. Females that carried both *yp1-tTA* and *tetO-hid* components died if raised on diet that lacked tetracycline. With the success of this genetic system in *D. melanogaster*, we isolated several yolk protein (*yp*) genes from *L. cuprina*^15^. Although a *yp* promoter was shown to direct high levels of a reporter gene in transgenic adult female *L. cuprina*, expression was not detected until the adult stage and only after a protein-meal. This was too late a stage to be useful for a building a TSS.

Alphey and colleagues developed an alternative single-component genetic system for making tephritid fruit fly TSS^16^. The system consists of a tetO-hsp70 enhancer-promoter driving expression of tTA transcripts that are sex-specifically spliced as they contain the regulated intron from the medfly *transformer* (*Cctra*) gene. Only the female transcript encodes tTA protein. If the TSS were raised on diet without tetracycline, autoregulation of tTA gene expression led to very high levels of tTA and female-specific lethality. The lethality is thought to be due to “transcriptional squelching” and happens at the late larval/pupal stages. To build *L. cuprina* TSS carrying a similar genetic system, we isolated and characterized the *tra* gene from *L. cuprina* and *C. hominivorax*^17^,^18^. Subsequently, *L. cuprina* TSS were made that overexpress the tetracycline dependent transactivator (tTA) in females when raised on diet without tetracycline^19^. To achieve very high levels of tTA expression it was necessary to employ a core promoter for a *L. cuprina hsp70* gene^20^. While the TSSs produced only males, female lethality was at the late larval/pupal stages, which is too late a stage for any appreciable savings in diet costs in a mass-rearing facility^21^.

Two groups have developed transgenic embryonic sexing systems (TESS) for *C. capitata* and the Caribbean fruit fly *Anastrepha suspensa*^22^,^23^. In these systems, tTA expression is driven by a promoter from a cellularization gene that is expressed in the early embryo. In the absence of tetracycline, tTA activates expression of *hid* transcripts, which are sex-specifically spliced as they contain the regulated *Cctra* intron. The *hid* transcripts encoded “phospho-mutated” versions of HID as the predicted MAPK phosphorylation sites had been changed to Alanine residues. In *D. melanogaster*, HID can be inhibited by MAPK phosphorylation in response to Ras-dependent growth signaling^24^. To develop *L. cuprina* TESS, we previously isolated and characterized the *L. sericata hid* gene and the promoter from the *L. sericata bottleneck* (*Lsbnk*) cellularization gene^25^. We found that widespread expression of *Lshid* was lethal in *D. melanogaster*. Further, we showed that the *Lsbnk* gene promoter drove expression of a GFP reporter gene in early *L. cuprina* embryos. Here we report the development of *L. cuprina* TESS that use the *Lsbnk* promoter to drive expression of tTA (*Lsbnk-tTA*), which activates expression of *Lshid* in the absence of tetracycline. The *Lshid* gene contains the sex-specific intron from the *C. hominivorax tra* gene. We found adult females carrying both components of the TESS needed to be fed a low dose of tetracycline in the first two days after eclosion in order to survive and lay eggs. This is most likely due to low levels of tTA expression in young adult females. To obtain only male offspring, TESS females were raised on diet without tetracycline from day 3 onwards. We show that female offspring of TESS raised under these conditions die at the embryo stage. We also report development of TESS where both *Lsbnk-tTA* and *tetO-Lshid* components are contained within a single gene construct. This is advantageous for transferring the technology to other calliphorid livestock pests and for “stabilizing” the transgene. The latter involves a two-step recombination/transposition procedure to effectively remove one of the *piggyBac* ends^26^,^27^.

## Results

### *tTAo* driven by the *Lsbnk* promoter is expressed in early embryos and other developmental stages

**Figure 1:**
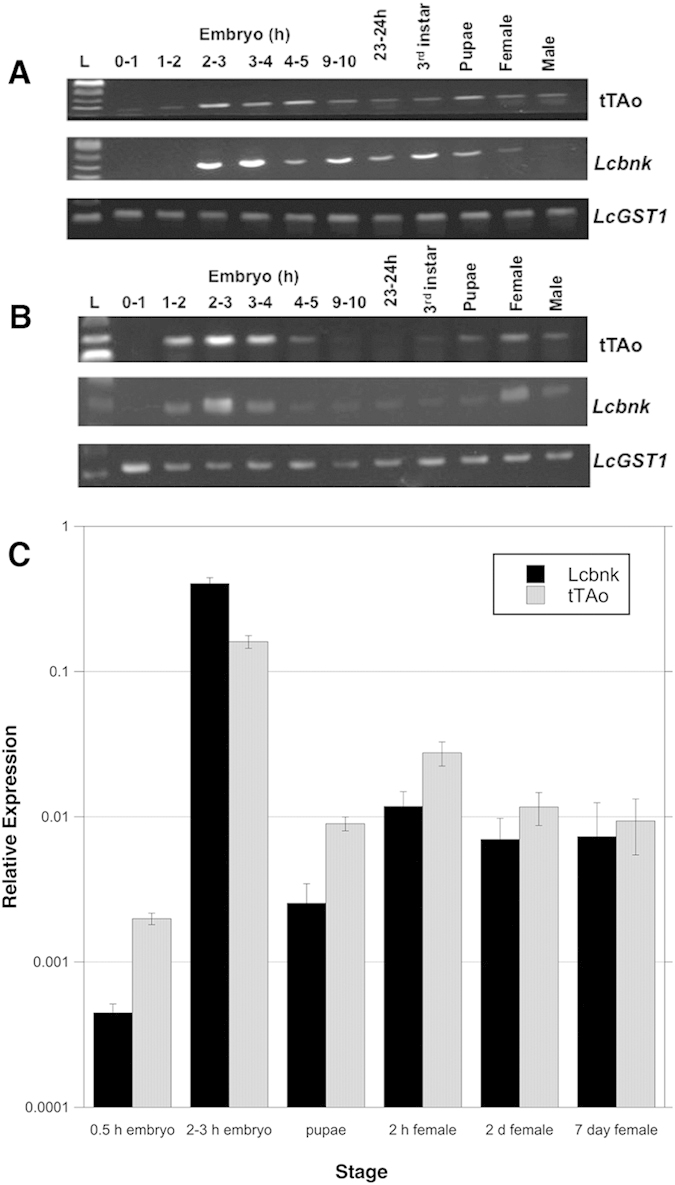
tTAo expression at different developmental stages. *tTAo* transcript levels in the DR2–6# (**A**) and DR2–7# (**B**) lines was analyzed by RT-PCR (34 cycles). The sizes of RT-PCR products are: 165 bp for *tTAo*, 203 bp for *Lcbnk* and 121 bp for *LcGST1.* (**C**) *Lcbnk* and *tTAo* relative expression levels at different developmental stages in the DR2#6 line as determined by quantitative RT-PCR. RNA levels are relative to the *LcGST1* reference gene. Mean and standard error from three measurements are shown.

The DR2 driver construct contains the *Lsbnk* promoter upstream of the *tTAo* coding region, which had been codon-optimized for translation in *L. cuprina*. The *Lsbnk-tTAo* gene cassette was in a *piggyBac* transformation vector containing a constitutively expressed ZsGreen marker gene. Nine *L. cuprina* transgenic lines were obtained by *piggyBac*-mediated germ-line transformation and bred to homozygosity. A preliminary RT-PCR analysis of RNA isolated from 2–3 h embryos found that most of the DR2 lines expressed similar levels of *tTAo* (data not shown). Two autosomal DR2 lines, #6 and #7, were selected for further analysis. RNA was isolated from pre-cellular embryos (0–2 h), embryos at cellularization (2–3 h) and after cellularization, first and third instar larvae, pupae and young adults. Pilot experiments were performed to identify the range of PCR cycles over which there was exponential amplification of *tTAo* and endogenous *L. cuprina bnk* (*Lcbnk*) from the cDNA templates. 34 PCR cycles was selected as amplification of *tTAo* and *Lcbnk* was within this range at early embryo timepoints and was detectable at later timepoints. The highest expression of *tTAo* and *Lcbnk* was around the time cells first form in developing *L. cuprina* embryos (2 h) (Fig. 1). No amplification products were detected from pre-cellular embryos (0–1 h), confirming there is no maternal expression. We also detected *tTAo* and *Lcbnk* expression at later developmental stages, notably in pupae and young adult females. As in previous studies, *L. cuprina glutathione S-transferase 1* (*LcGST1*) was employed as a reference gene^20^,^28^. We next measured the relative expression of *Lcbnk* and *tTAo* RNA levels in the DR2#6 line more accurately using quantitative RT-PCR (Fig. 1C). Expression levels were relative to *LcGST1*. The results were consistent with the semi-quantitative analysis. *Lcbnk* and *tTAo* RNA levels were highest in 2–3 h embryos. The *Lcbnk* transcript was more than 1000 times higher in 2–3 h embryos than pre-cellular 0–0.5 h embryos. Newly eclosed adult females (2 h) had the second highest level of *Lcbnk* RNA, but this was more than 30 times lower than at 2–3 h embryo. While *tTAo* RNA levels were highest at 2–3 h embryos, the level was only 6 times higher than in 2 h females. Further, *tTAo* RNA levels were generally higher than *Lcbnk* at all stages except at 2–3 h embryos. Thus it appears that *tTAo* driven by the *Lsbnk* promoter is not as tightly regulated in this line as the endogenous *Lcbnk* gene.

### Female-specific lethality of double heterozygous progeny from crosses of *Lsbnk*-*tTA* driver and *tetO*-*Lshid* effector lines

**Figure 2:**
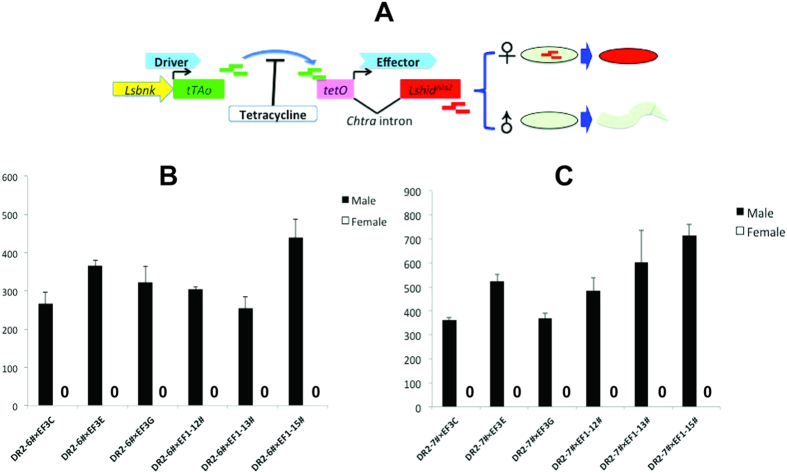
Female-specific lethality of double heterozygous lines. (**A**) Schematic illustration of the two-component system containing one driver and one effector. This system used an embryonic *Lsbnk* promoter to drive the tetracycline transactivator (*tTAo*), as well as a sex-specific intron (*Chtra*) splicing cassette linked to a cell death gene *Lshid*^Ala2^ lethal effector to achieve highly efficient female lethality early in the development of *L. cuprina.* Addition of tetracycline to the diet prevents activation of the *Lshid*^Ala2^ gene. 8 homozygous DR2–6# (**B**) or DR2–7# (**C**) males were crossed with 8 virgin females from homozygous effector lines and their offspring were raised on diet without tetracycline. The number of adult male and female offspring from each cross were counted. Error bars show the standard error of the mean (n = 3).

The EF1 and EF3 effector constructs both employ the *tetO*_*21*_*-Lchsp70* enhancer-promoter^19^ to control expression of *Lshid*. EF3 contains the wild type version of *Lshid* whereas EF1 has a phosphomutated version called *Lshid*^*Ala2*^, as the two conserved MAPK phosphorylation sites^25^ have been changed to encode Alanine. In both effector constructs, the sex-specific first intron from the *Chtra* gene was inserted within the *Lshid* coding region. The intron immediately follows the ATG translation start codon, as in the FL11 and FL12 tTA overexpression gene constructs used previously to make tetracycline-repressible female lethal lines of *L. cuprina*^19^. The sequence immediately 3′ of the intron in EF1 and EF3 is GCA, which differs slightly from GTC in FL11/12 and GTG in the calliphorid *tra* genes^18^. EF1 and EF3 also contained a constitutively expressed DsRedex2 marker gene and 5′ and 3′ *piggyBac* ends for transformation. Seven EF1 and seven EF3 transgenic *L. cuprina* lines were obtained by *piggyBac*-mediated transformation and were bred to homozygosity. In all lines the transgene had inserted onto one of the autosomes. In an initial screen, the DR2#6 line was crossed with homozygous EF1 and EF3 lines and the number of adult male and female offspring counted (Table S1). The expectation was that, in the absence of tetracycline, activation of *Lshid* gene expression in early embryos would lead to death of female offspring (Fig. 2A). Only females are predicted to die as only in females would *Lshid* transcripts be correctly spliced to produce mRNAs that encode LsHID protein. No female offspring were obtained from crosses with any of the EF1 lines (Table S1). Similarly, only male offspring were obtained from crosses with four of the EF3 lines (Table S1). Two of the EF3 lines produced a few female offspring. The weakest effector was EF3F as only approximately 50% of the females died. Thus it appears that, as expected^24^,^29^, the phosphomutated version of LsHID is more effective at promoting organismal death. This, however, would require confirmation with apoptosis assays. DR2 lines 6 and 7 and three each of the EF1 and EF3 lines that gave 100% dominant female lethality were selected for further analysis. Each cross was performed three times and the offspring counted. All DR2/EF1 and DR2/EF3 combinations produced 100% male offspring (Fig. 2B,C). That is, one copy of the driver and one copy of the effector gene was sufficient for 100% female lethality on diet that lacked tetracycline. Thus the genetic system appears to be very efficient at producing only male offspring.

### Female embryo-specific lethality of double homozygous lines

For mass rearing, it is necessary to have strains that are homozygous for driver and effector. Given that all selected driver and effector combinations appeared equally effective as double heterozygotes, we selected one driver line (#6), one EF1 line (#12) and one EF3 line (E) to make double homozygous lines. Larvae homozygous for driver and effector transgenes were selected based on intensity of fluorescence of the green and red fluorescent proteins. Double homozygous strain 1 (DH1) combines DR2#6 with EF1#12 whereas DH2 contains the same driver with EF3E. The DH strains were raised on diet supplemented with tetracycline (100 μg/mL). The DH strains have been maintained for at least 18 generations with no loss of green or red fluorescence intensity, confirming the accuracy of the initial selection of homozygous larvae. To induce female lethality in embryos, it is necessary to feed the parental generation diet without tetracycline^30^. If not, tetracycline is passed on from the mother to the eggs, which would prevent activation of *Lshid* expression in the embryo. However, we found that DH1 and DH2 produced very few eggs if the adults were not fed tetracycline. Further, an examination of the cages indicated that some of the females had died. Since DH1 and DH2 could be readily maintained on diet that contained tetracycline, this suggested that the female lethal system (Fig. 2A) had been activated in adults not fed tetracycline. This was presumably due to expression, albeit at a relatively low level, of *tTAo* in adult females in the DR2 lines (Fig. 1). We investigated the influence of tetracycline in the adult diet and female longevity more carefully by placing 50 pairs of the DH strains in a cage and daily counting the number of male and females (Fig. S1). If not fed tetracycline, DH females did not live as long as males. Further, half of the DH2 females died before the first egg collection (day 8). We then investigated if feeding adults a low level of tetracycline in water for the first few days after eclosion was sufficient to restore viability. For the DH1 line (Fig. S1C,D) and DH2 line (not shown), water with 10 μg/mL tetracycline for the first 2 days was sufficient to restore female viability. Subsequently, we found that a dose of 1 μg/mL for DH1 and 3 μg/mL tetracycline for DH2 for the first 2 days was sufficient for female viability and egg production on day 8 (not shown). The lower doses of tetracycline were preferred as it was less likely that mothers would pass on enough tetracycline to their offspring to inhibit tTA.

**Figure 3:**
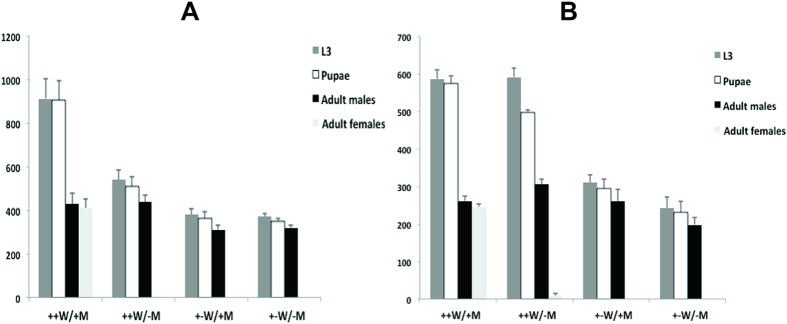
Female-specific lethality of DH1 (**A**) and DH2 (**B**) under different tetracycline conditions. Containers were set with 8 pairs of adults and the number of third instar larvae (L3), pupae and adult male and female offspring counted. ++W: water with 100 μg/mL tetracycline from day 1 (D1) to D8; +−W: water with limited tetracycline (1 μg/mL for DH1 and 3 μg/mL for DH2) from D1 to D2, then switched to water without tetracycline from D3 to D8; +M: ground meat (larval diet) with 100 μg/g tetracycline; -M: meat without tetracycline. Mean and standard error are shown from three replicate experiments.

With a limited tetracycline feeding regimen established under which DH females survived and laid eggs, we proceeded to determine if under these conditions female offspring died at the embryo stage. In all experiments, the larvae of the parental generation were raised on diet supplemented with a high dose of tetracycline (100 μg/mL). Firstly, we found that 100% of the female offspring died if adults of the parental generation were fed a limiting dose of tetracycline (1 or 3 μg/mL, days 0–2) and their offspring were raised on larval diet that had no tetracycline (Fig. 3, +−W/−M). Females were not rescued by adding tetracycline to the larval diet (Fig. 3, +−W/+M). This suggested that females were dying at the embryo stage or early larval stage. Importantly, females were fully viable if the parental generation and their larval offspring were fed diet that contained high levels of tetracycline (100 μg/mL) (Fig. 3, ++W, +M). Females also died if the parental generation was fed a high dose of tetracycline but the larval diet lacked tetracycline (Fig. 3, ++W/−M). Under these conditions we anticipated that females would survive if *tTAo* was only expressed in early embryos. That females did not survive suggested that *tTAo* was expressed during one or more of the larval stages. A low level of *tTAo* RNA was detected at the early first and wandering third instar larval stages (Fig. 1).

**Figure 4:**
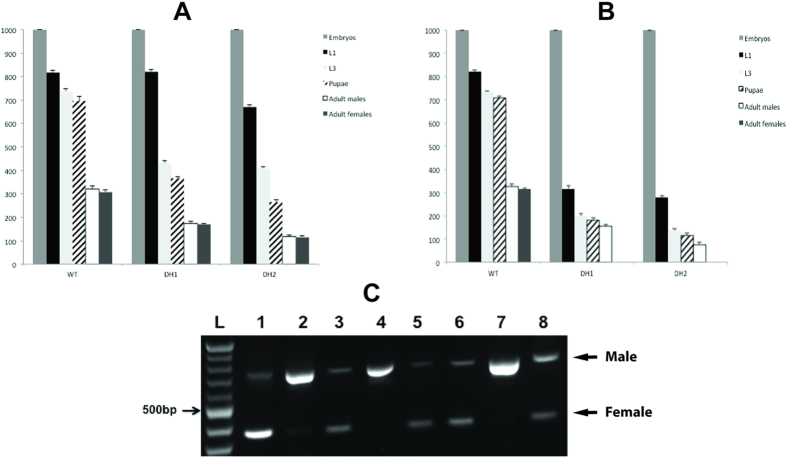
Determination of lethal stage in DH1 and DH2 lines. DH1, DH2 and wild-type (WT) flies reared on permissive (**A**) and restrictive tetracycline conditions (**B**). 1000 embryos were collected and the numbers of first instar larvae (L1), L3, pupae, adult males and adult females were recorded. Error bars show the standard error of the mean (n = 3). (**C**) Larval sex identification through detection of *Lctra* sex-specific splice variants. RNA was extracted from 100 embryos or L1 larvae and RT-PCR performed using a primer pair that detects the male (736 bp) or female- (325 bp) splice variants. Permissive tetracycline (++W, 100 μg/mL) and restricted tetracycline (+−W, 1 or 3 μg/mL for days 0–2) feeding regimen was as in the previous experiment (Fig. 3). Lane 1) WT♂: ♀ = 1; 2) WT♂: ♀ = 100; 3) 9–10 h embryos of DH1 under +−W condition; 4) L1 of DH1 under +−W condition; 5) L1 of DH1 under ++W condition; 6) 9–10 h embryos of DH2 under +−W condition; 7) L1 of DH2 under +−W condition; 8) L1 of DH2 under ++W condition; L DNA ladder.

The above analysis suggested that the female offspring of DH parents fed a diet with a low level of tetracycline (days 0–2) (+−W) died at the embryo stage. To confirm the stage of lethality, 1000 eggs were collected from the parental wild type strain, the DH1 and DH2 strains and the number of hatched first instar larvae, third instar larvae, pupae and adult males and females were counted. The strains were either fed a high (Fig. 4A) or limited level of tetracycline (Fig. 4B). The experiment was repeated three times. Equal numbers of adult males and females were obtained if the strains were fed high levels of tetracycline (Fig. 4A). The DH strains produced fewer adults per 1000 eggs than the parental strain. On limited tetracycline diet, no adult females were obtained from either DH strain (Fig. 4B). Importantly, approximately half as many first instar larvae were obtained on the limited tetracycline diet compared to the high tetracycline diet. This suggested that the DH females had died at the embryo stage. This was confirmed by RT-PCR analysis of RNA isolated from embryos at 9–10 h of development and hatched first instar larvae (Fig. 4C). The primers used amplify across the sex-specific first intron of the *L. cuprina tra* gene^17^. The male PCR product is larger due to incorporation of an additional exon. To calibrate the sensitivity of this assay, we first analyzed RNA isolated from adult male and female. cDNA was mixed at a 1:1 and 100:1 male:female ratio. As reported previously^17^, at a 1:1 ratio the smaller female product predominates (Fig. 4C, lane 1). At a 100:1 ratio the main product is from males. Nevertheless, the smaller female product was detected (Fig. 4C, lane 2). This suggested that if females made up 1% or more of the L1 population, their presence could be detected using this assay. Both male and female products were detected in 9–10 h embryos from DH1 (Fig. 4C, lane 3) and DH2 (Fig. 4C, lane 6) parents fed low levels of tetracycline (days 0–2). However, only the male product was detected in first instar larvae (Fig. 4C, lanes 4 and 7). Both male and female products were obtained from first instar larval RNA if the DH1 or DH 2 parents were fed a high level of tetracycline (Fig. 4C, lanes 5 and 8). This analysis confirms that the female offspring of the DH1 and DH2 strains died at the embryo stage.

### Female embryo-specific lethality of “All-in-one” lines

**Figure 5:**
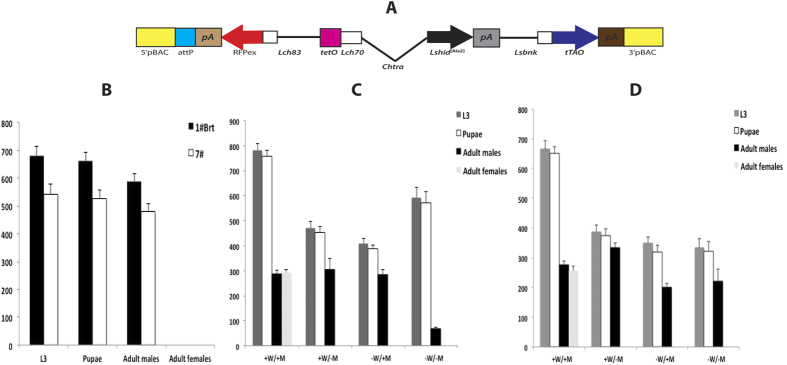
Female-specific lethality of All-in-one lines. (**A**) Schematic illustration of the All-in-one construct, which contains both driver and effector cassettes in the pB[Lshsp83-RFPex] vector. (**B**) Female-specific lethality of heterozygotes. 8 homozygous 1#Brt or 7# (X-linked) virgin females were crossed with 8 wild type males and their offspring were raised on diet without tetracycline. The number of L3, pupae, adult male and female offspring from each cross were counted. Female-specific lethality of homozygous lines 1#Brt (**C**) and 7# (**D**). Parental generation feeding regimen: +W: water with 100 μg/mL tetracycline from D1 to D8; −W: water with no tetracycline from D1 to D8. +M is 100 μg/g tetracycline added to the larval diet (meat), −M is without tetracycline. Mean and standard error are shown from three replicate experiments.

**Figure 6:**
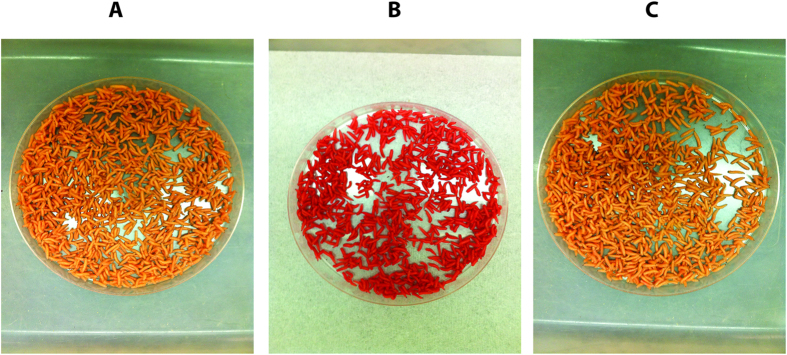
Sex-specific overexpression of the fluorescent marker gene in transgenic *L. cuprina* All-in-one-1#Brt line. The presence of tetracycline turned off the lethal system and the marker gene expression is due only to the basal activity of the *Lchsp83* promoter (**A**). In the absence of tetracycline, females died early due to expression of *Lshid*^*Ala2*^, while males overexpress the red marker gene DsRedex2 (RFPex) (**B**). Supply of tetracycline in the larval diet blocks the overexpression of RFPex (**C**).

One of our long-term aims is to transfer the technology for making TESS to other calliphorid livestock pests, notably *C. hominivorax*. This would be simpler if the two components could be successfully combined into a single gene construct, as that would reduce the number of transgenic lines that need to be made and analyzed. Consequently, we inserted the *Lsbnk*-*tTAo* driver and *tetO*_*21*_*-Lshid*^*Ala2*^ effector into a *piggyBac* transformation vector with the DsRedex2 marker used previously (Fig. 5A). Four transgenic *L. cuprina* lines were obtained with this “All-in-one” construct by *piggyBac*-mediated germ-line transformation. Two lines, 1#Brt and 7#, were homozygous viable and fertile. In the 7# line, the transgene had inserted onto the X chromosome, whereas the 1#Brt line is autosomal. The homozygous lines have been maintained for at least 12 generations on a high dose of tetracycline (100 μg/mL) with no loss of fluorescence intensity. Based on our previous experience with the DH1 and DH2 strains, we anticipated that adult females would not be fully viable and fertile unless fed tetracycline for the first two days after eclosion. However, we found that for both All-in-one lines, homozygous females did not need to be fed water supplemented with tetracycline. To determine if the All-in-one lines were as efficient as the DH strains, homozygous 1#Brt and 7# females not fed tetracycline were crossed with males from the parental wild type strain and offspring counted (Fig. 5B). Both All-in-one lines produced 100% males. Thus, for both All-in-one lines, one copy of the transgene is sufficient for 100% female lethality, suggesting the genetic system is as efficient as in the DH strains. We next counted the offspring of homozygous 1#Brt and 7# lines raised with or without tetracycline (Fig. 5C,D). If tetracycline was added to the parental and larval diets (+W/+M), both lines produced approximately equal numbers of adult males and females. As expected, in the absence of tetracycline (−W/−M), both lines only produced adult males. However, the pupal hatch rate was less than anticipated, particularly for the 1#Brt line (Fig. 5C). This suggested that males were dying in the absence of tetracycline. This was puzzling since the genetic system was designed to be lethal only to females. In addition to a low pupal hatch rate, we also observed that wandering third instar larvae expressed very high levels of the DsRed2ex marker gene (Fig. 6B). Overexpression of the marker gene had been previously observed in female larvae that overexpress tTA^19^. The explanation was that tTA bound to tetO stimulates transcription of both the linked marker gene and tTA. The overexpression of the marker gene in the All-in-one larvae suggested that tTA bound to tetO is increasing expression of the linked marker gene and possibly also *tTAo* driven by the *Lsbnk* promoter. If so, males could be dying due to expression of high levels of tTA. If this explanation was correct, males should be rescued by addition of tetracycline to the larval diet. Indeed, we observed a much higher pupal hatch rate for the 1#Brt line if the larval diet contained tetracycline (−W/+M). Under these feeding conditions, no female offspring were obtained. Consistent with our interpretation, larvae fed tetracycline did not overexpress the marker gene (Fig. 6C). Interestingly, addition of high levels of tetracycline to the parental but not larval diet (+W/−M) was also sufficient to rescue 1#Brt males (Fig. 5C). Presumably, this is providing enough tetracycline to prevent tTA accumulating to toxic levels in male larvae. However, female lethality was 100%, suggesting there is sufficient *tTAo* expression in larvae to activate *Lshid* transcription.

**Figure 7:**
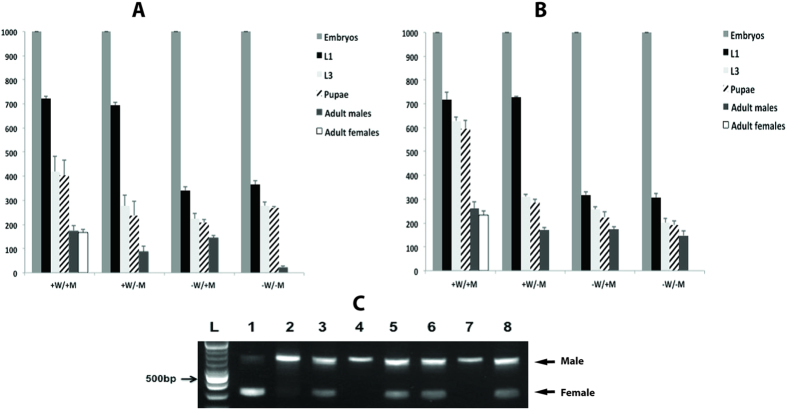
Determination of the stage of female lethality for All-in-one lines. Staged lethality of homozygous lines 1#Brt (**A**) and 7# (**B**) were tested under different tetracycline conditions. (**C**) Larval sex identification through detection of *Lctra* sex-specific splice variants. Lane 1) WT♂: ♀ = 1; 2) WT♂: ♀ = 100; 3) 9–10 h embryos of 1#Brt under -W condition; 4) L1 of 1#Brt under -W condition; 5) L1 of 1#Brt under +W condition; 6) 9–10 h embryos of 7# under −W condition; 7) L1 of 7# under −W condition; 8) L1 of 7# under +W condition; L DNA ladder.

We next investigated if females from the All-in-one lines died at the embryo stage when the parental diet lacked tetracycline. The approach was the same as used previously with the DH strains (Fig. 4). If the parents were fed high levels of tetracycline in water (100 μg/mL), approximately 70–75% of the embryos from the 1#Brt and 7# lines hatched into first instar larvae (Fig. 7A,B, +W/+M and +W/−M). In contrast, if the parents were not fed tetracycline only about 35% of the embryos hatched into first instar larvae. This suggested that the females were dying as embryos. Molecular analysis confirmed that the hatched first instar larvae were male (Fig. 7C lanes 4 and 7). As observed in the previous experiment, the male pupal hatch rate for the 1#Brt was low unless the larval diet contained tetracycline (Fig. 7A, −W/−M compared to –W/+M). One notable difference between the two All-in-one lines is that on diet supplemented with tetracycline (+W/+M), approximately 400 pupae were obtained from 1000 eggs for the 1#Brt line, whereas about 600 pupae were obtained from the 7# line (Fig. 7A,B).

## Discussion

For an SIT program, a TSS offers several potential advantages compared to a wild type bisexual strain. These include:

1. More efficient population suppression in the field with male-only release^10^,^11^.
2. Considerable savings in diet costs and distribution costs. The former is only if the females die at the embryo or very early larval stages.
3. The capacity of the mass-rearing plant is essentially doubled. For the *C. hominivorax* SIT program this could be important if sterile flies are needed to both maintain the buffer zone near Colombia and to control an outbreak in any of the countries now free of the pest.
4. The fluorescent marker could provide an easy means for distinguishing the released sterilized flies from wild type flies caught in the field^13^.
5. Fertile males could be released if female-lethality is dominant. Eliminating the sterilization step should improve the fitness of the released males^31^. Moreover, modeling suggests that a fertile male release could be more effective than SIT, particularly if the males carry multiple conditional female lethal transgenes^32^,^33^.

The previously developed *L. cuprina* TSS had all of these advantages except that females died at the pupal stage^19^. To address this issue, we have taken an approach used previously with tephritid fruit flies^22^,^23^ to develop *L. cuprina* TESS. The initial TESS made were double homozygous for a *tTAo* (codon-optimized tTA) driver and *Lshid* effector. The promoter from the *Lsbnk* cellularization gene was used to drive *tTAo* expression. *tTAo* RNA levels were highest around the time of cellularization in the early embryo. However, *tTAo* was also detected at later stages of development, notably newly eclosed adult females. This expression pattern could explain why double homozygous females were not fully viable and fertile unless fed a low dose of tetracycline for the first two days after eclosion. Adults could then be reared on diet without added tetracycline and eggs collected 8 days after eclosion. Under these dietary conditions, we showed that the female offspring of the TESS died at the embryo stage. We don’t anticipate that such a tetracycline-feeding regimen would be difficult to implement at a mass-rearing facility. Nevertheless, the TESS could potentially be improved by using a different promoter to drive tTA expression. Ideally the promoter would have high activity in the embryo at around the time of cellularization but little to no activity at other stages of development. The recent completion of a draft *L. cuprina* genome sequence^34^ will facilitate the isolation of promoters for other cellularization genes. Alternatively, the *Lshid* effector could be modified to be less responsive to tTA by using fewer copies of the tetO binding site and a weaker core promoter. The tetO_21_-*Lchsp70* enhancer-promoter was chosen to make the *Lshid* effectors as this was very effective in the tTA overexpression systems developed previously in *L. cuprina*^19^. The double homozygous strains are not ideal for a fertile release program as the two components would segregate independently in subsequent generations.

*L. cuprina* TESS were also made with an All-in-one construct that contained the *Lsbnk-tTAo* driver and *tetO*_*21*_*-Lshid* effector in one genetic system. An All-in-one system is attractive at it could reduce the number of transgenic lines that need to be made and analyzed. This would make it easier to transfer the TESS technology to other calliphorid livestock pests such as *C. hominivorax*, the Old World screwworm, *Chrysomya bezziana* and the European green blowfly *L. sericata*. It would also be easier to “stabilize” a TSS that contained a single transgene. Stabilization involves a two-step recombination/transposition procedure that removes one or both of the *piggyBac* ends^26^,^27^,^35^. This could improve stability under mass rearing if *L. cuprina* express a transposase that recognizes the *piggyBac* ends. However, *piggyBac* was not found in the *L. cuprina* genome sequence nor were we able to detect *piggyBac* in *L. cuprina* genomic DNA by Southern DNA hybridization analysis (J. Heinrich and M. Scott, unpublished observations). Excision of a *piggyBac* end would also diminish the potential for horizontal transfer of the transgene. With an All-in-one system it would also be easier to build strains with multiple female-lethal transgenes, which modeling suggests could be particularly effective in a fertile male release program^32^,^33^. However, the specific All-in-one system described in this study would not be suitable for fertile male releases as we found that males needed to be fed tetracycline in the larval diet. That is, a fertile release program is predicted to be more efficient than SIT if the female lethal trait is passed on through viable and fertile male offspring. Male lethality was most likely due to high levels of expression of tTA, as we observed overexpression of the linked marker gene and male viability was restored by adding tetracycline to the diet. The All-in-one construct could be potentially modified to prevent tTA from accumulating to high levels. For example, by bracketing the *tetO-Lshid* transgene with chromatin insulators used previously in *piggyBac* transformation vectors^36^. Of the two All-in-one lines made, 7# would appear to be more promising for mass rearing as this line produced more pupae from 1000 eggs on diet supplemented with tetracycline. Pupal production per gram of eggs is one of the most important biological parameters in the *C. hominivorax* mass rearing facility^21^. In addition, it will be necessary to determine if other important fitness parameters (e.g. pupal weight, embryo hatch frequency) and male mating competitiveness are comparable to the parental wild type strain. Surprisingly, unlike the double homozygous strains, females from the All-in-one lines did not need to be fed tetracycline for the first two days after eclosion. This suggests that the All-in-one adult females express lower levels of the LsHID^Ala2^ protein. This could be due to position effects in the two lines that were homozygous viable or could reflect a property of the All-in-one construct. In any case, the results from the *L. cuprin*a All-in-one lines are sufficiently encouraging that we plan to evaluate this system in other calliphorid pests including *C. hominivorax*.

## Methods

### Fly rearing and germ-line transformation

The LA07 wild type strain of *L. cuprina* was maintained as previously described^19^. *L. cuprina* transformation was as recently described^19^, with embryos microinjected with a mixture of synthesized *piggy*Bac RNA helper (300 μg/mL), *Lchsp83*-pBac helper (200 μg/mL) and one of pBac[Driver], pBac[Effector] or pBac[All-in-one] plasmid (700 μg/mL). First instar larvae showing transient expression of the ZsGreen or DsRedex2 marker were selected and raised on raw ground beef. G_0_ adults were crossed to wild type flies and offspring screened for expression of the fluorescent marker at late embryo/first instar stages. Homozygous individuals were selected at the wandering third instar larval stage based on fluorescence intensity and bred to create a stable line. The chromosomal insertion site of a transgene was determined by inverse PCR with genomic DNA templates that had been digested with MboI and TaqI as described previously^37^.

### Driver and effector plasmid construction

The general strategy was to assemble the driver or effector gene cassette in the cloning vector pBluescript II KS (–) and then excise the gene cassette by digestion with XhoI and NotI and clone into the unique XhoI and PspOMI sites in the *piggy*Bac transformation vectors pB [*Lchsp83*-ZsGreen]^38^ or pB[*Lchsp83*-DsRedex2]^19^. To assemble the driver construct, firstly tTA was excised from a tetO_21_-Dmh70-tTA-SV40 plasmid made previously^19^ and replaced with tTAo that was codon-optimized for expression in *L. cuprina* (synthesized by Genscript). A 860 bp DNA fragment containing the *Lsbnk* gene promoter^25^ was obtained by PCR amplification from *L. sericata* genomic DNA using the *Lsbnk*-Prom-F and *Lsbnk*-Prom-R primers (Table S2) and cloned into pGEM-T (Promega). After confirmation of the nucleotide sequence of the *Lsbnk* promoter, the tetO_21_-Dmh70 enhancer-promoter was excised and replaced with the *Lsbnk* promoter. Then the *Lsbnk* -tTAo-SV40 cassette was excised and ligated with the pB [*Lchsp83*-ZsGreen].

To assemble the effector construct, the *Lshid* CDS and part of the 5’ UTR^25^ was amplified from *L. sericata* embryo cDNA using primers Lshid-5UTR-F and Lshid-R and cloned into pGEM-T for nucleotide sequence confirmation. A 846 bp fragment containing the SV40 polyA signal was obtained by amplification using the primers SV40-BgIII-F and SV40-XhoI-R (Table S2) and pBS-FL3^19^ plasmid template and then ligated back to pBS-FL3 using unique HindIII and XhoI sites. The modified plasmid, pBS-FL3-1, was used as a template for PCR with the primer pair NcoIATGGCGNWStra and NWStra-SpeI. The 645 bp fragment obtained was digested with NcoI and SpeI and ligated with pBS-FL3-1 that had been digested with the same enzymes. Then a 1309 bp *Chtra* intron fragment was amplified from pBS-FL3-1 using primers NSWtra-StuI and Lshidaa2-NSWtra, and a 966 bp *Lshid* fragment was amplified from pGEM-*Lshid* using primers NWStra-Lshidaa2 and LshidTGA-BgIII. The fragments were purified and combined together as template for PCR with the primer pair NWStra-Lshidaa2 and LshidTGA-BgIII. The 2217 bp *Chtra* intron-ATG-*Lshid* fragment obtained was inserted into pBS-FL3-1 at the unique StuI and BgIII sites to generate pBS-tetO21-Dmhsp70-Chtra ATG-Lshid-SV40. A 921 bp fragment of *Lshid* was excised by digestion with NWStra-LshidAla2 and LshidTGA-BgIII and replaced with a synthetic fragment (Genscript) with two alanine mutations (LsHID^Ala2^). To replace the *Dmhsp70* core promoter with *Lchsp70* core promoter in pBS-effector plasmids, a 1991 bp fragment containing the *Lchsp70* promoter and *Chtra* intron was cut from pBS-FL11^19^ and inserted into all pBS-effector plasmids using unique BamHI and StuI sites. Finally, effector cassettes were excised from pBS and ligated with the pB[Lchsp83-DsRedex2]. To assemble the all-in-one construct, a 667 bp fragment containing the P10 polyA site was amplified from pB [DmPub-DsRed-P10] using P10pA-F and P10pA-R and ligated to pBS-Lsbnk-tTAo-SV40 using unique XhoI and HindIII sites. Then the Lsbnk-tTAo-P10 gene cassette was excised using SaII and XhoI and ligated to the effector construct that had been digested with XhoI.

### Female lethality test and embryo-specific lethality assessments

For the two-component system, homozygous virgin females from the effector lines were crossed with homozygous males from driver lines to generate double heterozygous female-specific embryonic lethal strains. The double heterozygous strains were inbred and their progeny screened to select only individuals homozygous for both the driver and effector construct (double homozygous) by epifluorescence microscopy based on fluorescence intensity. Lines were maintained on diet supplemented with 100 μg/mL tetracycline. To assess female lethality in a double heterozygous condition, 8 newly emerged males from a homozygous driver line and 8 newly emerged virgin females from a homozygous effector line were put in one bottle and kept on tetracycline-free adult diet for 8 days. Meat was provided for oviposition and embryos collected 24 h later. Larvae were reared on tetracycline-free raw ground beef, pupae collected and the number of adult males and females were counted. Female lethality in a double homozygous condition was addressed in the same way except newly emerged adults were fed water containing tetracycline (1 μg/mL for DH1 or 3 μg/mL for DH2) for the first 2 days and then switched to a tetracycline free diet. Embryos were collected as previously and larvae were reared on free or 100 μg/g tetracycline raw ground beef (90/10) and the number of 3^rd^ instar larvae, pupae, adult males and females were counted. To assess embryo-specific lethality, embryos were collected on ground beef then transferred to moist black filter paper in a petri dish and counted. Each petri dish held 1000 embryos. The number of hatched first instar larvae were counted and then transferred to meat. The number of 3^rd^ instar larvae, pupae, adult males and females were monitored. All lethality tests were done in triplicate.

To assess female lethality in a heterozygous condition for the All-in-one construct, 8 homozygous virgin females and 8 wild type males were put in one bottle and kept on tetracycline-free adult diet for 8 days. The embryos were collected as previously described and larvae were raised on diet without tetracycline. Then the number of 3^rd^ instar larvae, pupae, adult males and females were counted. Female lethality in a homozygous condition was addressed in the same way except newly emerged adults were fed water without or with 100 μg/mL tetracycline, then embryos were collected and larvae were reared without or with 100 μg/g tetracycline raw ground beef. The embryo-specific lethality was addressed as previously described.

### Determination of tTAo expression and larval sex by RT-PCR and qRT-PCR analysis

To compare relative *tTAo* expression at different developmental stages in homozygous driver lines DR2–6# and DR2–7#, the total RNA was extracted at different time points using the RNeasy® Mini Kit (QIAGEN) according to the manufacturer’s instructions. Isolated RNA was subsequently treated with RNase-Free DNase Set (QIAGEN). 5 μg RNA was used to synthesize cDNA using Superscript III First Strand Synthesis Supermix (Invitrogen) following the manufacturer’s instructions. A control reaction lacking Superscript III was included for each treatment. PCR reactions were assembled using Advantage 2 Polymerase Mix (Clontech) and subjected to the following thermal cycling parameters: initial denaturation for 3 min at 94 °C, 34 cycles of (94 °C for 30 s, 52 or 60 °C for 30 s, 72 °C for 1 min), final extension for 5 min at 72 °C. The primer pairs used were tTAo-qRT-F and tTAo-qRT-R (Table S2).

To more accurately measure relative expression levels, qRT-PCR was performed with an Applied Biosystems ABI7900HT Fast Real-time PCR system and Maxima SYBR Green/RoxqPCR Master Mix (Fermentas). All primer sets were tested with standard PCR, and run on a gel to confirm amplification of the desired size products. Samples were assayed in triplicate in a 12.5 μL final volume containing 1 μL (less than 100 ng) of cDNA (–RT was used as negative control for each line), 1× Maxima SYBR Green/RoxqPCR Master Mix (Fermentas), and 0.3 μM each primer and subjected to the following thermal cycling parameters: initial denaturation for 10 min at 95 °C, 40 cycles of (95 °C for 15 s, 60 °C for 30 s, 72 °C for 30 s), dissociation curve analysis at (72 °C for 30 s). The primer pairs used were tTAo-qRT-F/tTAo-qRT-R, Lcbnk-qRT-F/ Lcbnk-qRT-R and LcGST1-F/ LcGST1-R (Table S2). C_t_ values were called and averaged for each triplicate by SDS software (version 2.4). Samples were normalized to *LcGST1* and relative expression levels were calculated using the formula 2^−∆∆Ct^. Graphs were generated using Microsoft Excel.

To determine sex, RT-PCR was performed using an *Lctra* primer pair, Lctra-F/LctraR-qRT, and the following thermal cycling parameters: initial denaturation for 3 min at 94°C, 34 cycles of (94 °C for 30 s, 54 °C for 30 s, 72°C for 1 min), final extension for 5 min at 72 °C.

### Statistical analysis

The Chi-square test was used to statistically analyze the +/− tetracycline viability data (number flies, not ratios) using SAS JMP program.

## Additional Information

**Accession numbers**: The GenBank accession numbers for the plasmids developed in this study are: pDR2: KT749916, pEF1: KT749917, pEF3: KT749918, pAll-in-one: KT749919.

**How to cite this article**: Yan, Y. and Scott, M. J. A transgenic embryonic sexing system for the Australian sheep blow fly *Lucilia cuprina*. *Sci. Rep.* **5**, 16090; doi: 10.1038/srep16090 (2015).

## Supplementary Material

Supplementary Information

## References

[b1] Sandeman R. M. *et al.* Control of the sheep blowfly in Australia and New Zealand–are we there yet? Int. J. Parasitol. 44, 879–891 (2014). 25240442 10.1016/j.ijpara.2014.08.009

[b2] Wyss J. H. Screwworm eradication in the Americas. Ann. N. Y. Acad. Sci. 916, 186–193 (2000). 11193620 10.1111/j.1749-6632.2000.tb05289.x

[b3] Vargas-Teran M., Hofmann H. C. & Tweddle N. E. in Sterile Insect Technique. Principles and Practice in Area-Wide Integrated Pest Management (eds Dyck V. A., Hendrichs J. & Robinson A. S.) 629–650 (Springer, 2005).

[b4] Foster G. G., Weller G. L., James W. J., Paschalidis K. M. & McKenzie L. J. in *Management of insect pests: nuclear and related molecular and genetic techniques*. 299-312 (International Atomic Energy Agency, 1993).

[b5] Stevens J. R. & Wallman J. F. The evolution of myiasis in humans and other animals in the Old and New Worlds (part I): phylogenetic analyses. Trends Parasitol. 22, 129–136 (2006). 16459148 10.1016/j.pt.2006.01.008

[b6] Alexander J. L. Screwworms. J. Am. Vet. Med. Assoc. 228, 357–367 (2006). 16448356 10.2460/javma.228.3.357

[b7] Knipling E. F. Possibilities of insect control or eradication through the use of sexually sterile males. J. Econ. Entomol. 48, 459–462 (1955).

[b8] Mahon R. J. in FLICS: flystrike & lice IPM control strategies: *proceedings of a national conference.* (eds B. Horton & S. C. Champion) 225-232 (Tasmanian Institute of Agricultural Research, University of Tasmania, 2001).

[b9] Franz G. in Sterile Insect Technique. Principles and Practice in Area-Wide Integrated Pest Management (eds Dyck V. A., Hendrichs J. & Robinson A. S.) 427–451 (Springer, 2005).

[b10] McInnis D. O., Tam S., Grace C. & Miyashita D. Population suppression and sterility induced by variable sex ratio, sterile insect releases of *Ceratitis capitata* (Diptera: Tephritidae) in Hawaii. Ann. Entomol. Soc. Am. 87, 231–240 (1994).

[b11] Rendon P., McInnis D., Lance D. & Stewart J. Medfly (Diptera: Tephritidae) genetic sexing: large-scale field comparison of males-only and bisexual sterile fly releases in Guatemala. J. Econ. Entomol. 97, 1547–1553 (2004). 15568342 10.1603/0022-0493-97.5.1547

[b12] Alphey L., Nimmo D., O’Connell S. & Alphey N. Insect population suppression using engineered insects. Adv. Exp. Med. Biol. 627, 93–103 (2008). 18510017 10.1007/978-0-387-78225-6_8

[b13] Scott M. J., Morrison N. I. & Simmons G. S. in Transgenic Insects: Techniques and Applications (ed Benedict M.) Ch. 10, 152–167 (CABI, 2014).

[b14] Heinrich J. C. & Scott M. J. A repressible female-specific lethal genetic system for making transgenic insect strains suitable for a sterile-release program. Proc Natl Acad Sci USA 97, 8229–8232 (2000). 10890889 10.1073/pnas.140142697 PMC26929

[b15] Scott M. J. *et al.* Organisation and expression of a cluster of *yolk protein* genes in the Australian sheep blowfly, *Lucilia cuprina*. Genetica 139, 63–70 (2011). 20844939 10.1007/s10709-010-9492-6

[b16] Fu G. *et al.* Female-specific insect lethality engineered using alternative splicing. Nature Biotechnol. 25, 353–357 (2007). 17322873 10.1038/nbt1283

[b17] Concha C. & Scott M. J. Sexual development in *Lucilia cuprina* (Diptera, Calliphoridae) is controlled by the *transformer* gene. Genetics 182, 785–798 (2009). 19433631 10.1534/genetics.109.100982 PMC2710159

[b18] Li F., Vensko S. P. 2nd, Belikoff E. J. & Scott M. J. Conservation and Sex-Specific Splicing of the *transformer* Gene in the Calliphorids *Cochliomyia hominivorax*, *Cochliomyia macellaria* and *Lucilia sericata*. PLoS One 8, e56303 (2013). 23409170 10.1371/journal.pone.0056303 PMC3567074

[b19] Li F., Wantuch H. A., Linger, R. J., Belikoff E. J. & Scott M. J. Transgenic sexing system for genetic control of the Australian sheep blow fly *Lucilia cuprina*. Insect Biochem. Mol. Biol. 51, 80–88 (2014). 24928635 10.1016/j.ibmb.2014.06.001

[b20] Concha C. *et al.* Organization and expression of the Australian sheep blowfly (*Lucilia cuprina*) *hsp23*, *hsp24*, *hsp70* and *hsp83* genes. Insect Mol. Biol. 21, 169–180 (2012). 22506286 10.1111/j.1365-2583.2011.01123.x

[b21] Chen H., Chaudhury M. F., Sagel A., Phillips P. L. & Skoda S. R. Artificial diets used in mass production of the New World screwworm, *Cochliomyia hominivorax*. J. Appl. Entomol. 138, 708–714 (2014).

[b22] Schetelig M. F. & Handler A. M. A transgenic embryonic sexing system for *Anastrepha suspensa* (Diptera: Tephritidae). Insect Biochem. Mol. Biol. 42, 790–795 (2012). 22858603 10.1016/j.ibmb.2012.07.007

[b23] Ogaugwu C. E., Schetelig M. F. & Wimmer E. A. Transgenic sexing system for *Ceratitis capitata* (Diptera: Tephritidae) based on female-specific embryonic lethality. Insect Biochem. Mol. Biol. 43, 1–8 (2013). 23137881 10.1016/j.ibmb.2012.10.010

[b24] Bergmann A., Agapite J., McCall K. & Steller H. The *Drosophila* gene *hid* is a direct molecular target of *Ras*-dependent survival signaling. Cell 95, 331–341 (1998). 9814704 10.1016/s0092-8674(00)81765-1

[b25] Edman R. M. *et al.* Functional characterization of calliphorid cell death genes and cellularization gene promoters for controlling gene expression and cell viability in early embryos. Insect Mol. Biol. 24, 58–70 (2015). 25225046 10.1111/imb.12135

[b26] Horn C. & Handler A. M. Site-specific genomic targeting in *Drosophila*. Proc. Nat. Acad. Sci. USA 102, 12483–12488 (2005). 16116081 10.1073/pnas.0504305102 PMC1194931

[b27] Schetelig M. F. *et al.* Site-specific recombination for the modification of transgenic strains of the Mediterranean fruit fly *Ceratitis capitata*. Proc Natl Acad Sci USA 106, 18171–18176 (2009). 19828439 10.1073/pnas.0907264106 PMC2775304

[b28] Bagnall N. H. & Kotze A. C. Evaluation of reference genes for real-time PCR quantification of gene expression in the Australian sheep blowfly, Lucilia cuprina. Med. Vet. Entomol. 24, 176–181 (2010). 20604863 10.1111/j.1365-2915.2010.00866.x

[b29] Schetelig M. F., Nirmala X. & Handler A. M. Pro-apoptotic cell death genes, *hid* and *reaper*, from the tephritid pest species, *Anastrepha suspensa*. Apoptosis 16, 759–768 (2011). 21630017 10.1007/s10495-011-0610-4

[b30] Horn C. & Wimmer E. A. A transgene-based, embryo-specific lethality system for insect pest management. Nat. Biotechnol. 21, 64–70 (2003). 12483222 10.1038/nbt769

[b31] Crystal M. M. Sterilization of screwworm flies (Diptera: Calliphoridae) with gamma rays: restudy after two decades. J. Med. Entomol. 15, 103–108 (1979).

[b32] Thomas D. D., Donnelly C. A., Wood R. J. & Alphey L. S. Insect population control using a dominant, repressible, lethal genetic system. Science 287, 2474–2476 (2000). 10741964 10.1126/science.287.5462.2474

[b33] Schliekelman P. & Gould F. Pest control by the release of insects carrying a female-killing allele on multiple loci. J. Econ. Entomol. 93, 1566–1579 (2000). 11142283 10.1603/0022-0493-93.6.1566

[b34] Anstead C. A. *et al.* *Lucilia cuprina* genome unlocks parasitic fly biology to underpin future interventions. Nature communications 6, 7344 (2015). 10.1038/ncomms8344 PMC4491171 26108605

[b35] Dafa’alla T. H. *et al.* Transposon-free insertions for insect genetic engineering. Nat. Biotechnol. 24, 820–821 (2006). 16823373 10.1038/nbt1221

[b36] Sarkar A. *et al.* Insulated *piggyBac* vectors for insect transgenesis. BMC Biotechnol. 6, 27 (2006). 16776846 10.1186/1472-6750-6-27 PMC1525164

[b37] Li X., Heinrich J. C. & Scott M. J. *piggyBac*-mediated transposition in *Drosophila melanogaster*: an evaluation of the use of constitutive promoters to control transposase gene expression. Insect Mol. Biol. 10, 447–455 (2001). 11881809

[b38] Concha C. *et al.* Efficient germ-line transformation of the economically important pest species *Lucilia cuprina* and *Lucilia sericata* (Diptera, Calliphoridae). Insect Biochem. Mol. Biol. 41, 70–75 (2011). 20869440 10.1016/j.ibmb.2010.09.006

