# Author Correction: A transgenic embryonic sexing system for the Australian sheep blow fly *Lucilia cuprina*

**DOI:** 10.1038/s41598-020-63126-0

**Published:** 2020-04-20

**Authors:** Ying Yan, Maxwell J. Scott

**Affiliations:** Department of Entomology, North Carolina State University, Campus Box 7613, Raleigh, NC 27695-7613 USA

Correction to: *Scientific Reports* 10.1038/srep16090, published online 05 November 2015

A supplementary file containing Figure S1 is missing from this Article. Figure S1 and its accompanying legend are provided below as Fig. 1.

**Figure 1:**
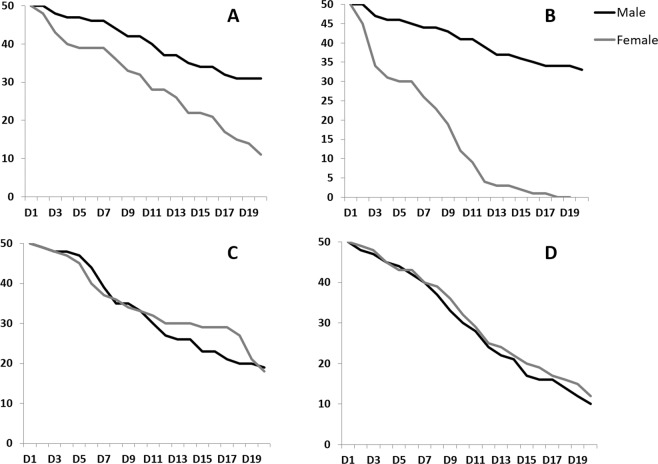
Adult longevity when raised on diet without or with limited tetracycline. For all tests the cages contained 50 adult males and 50 adult females at day 0. (**A**) DH1 on diet without tetracycline; (**B**) DH2 on diet without tetracycline; (**C**) DH1 on limited tetracycline (10 ug/ml for 2 d); (**D**) DH1 on limited tetracycline (10 ug/ml for 4d).

