# Supplementary material for: A transgenic embryonic sexing system for the Australian sheep blow fly Lucilia cuprina
Source: Sci Rep. 2015 Nov 5;5:16090. doi: 10.1038/srep16090 (PMC4633611; doi:10.1038/srep16090)
Supplement: Supplementary Information [file srep16090-s1.doc]

## Supplementary Information

A transgenic embryonic sexing system for the Australian sheep blow fly *Lucilia cuprina*

Ying Yan and Maxwell J. Scott.

Department of Entomology, North Carolina State University, Campus Box 7613, Raleigh, NC, 27695-7613

**Table S1.** Lethality test data from crosses of the DR2#6 line with effector lines.

| **Effector** | **Line** | **Pupae** | **Male** | **Female** | **%Male** |
| --- | --- | --- | --- | --- | --- |
| EF1 | 1 | 329 | 234 | 0 | 100％ |
|  | 8 | 125 | 72 | 0 | 100％ |
|  | 12 | 364 | 316 | 0 | 100％ |
|  | 13 | 370 | 314 | 0 | 100％ |
|  | 14 | 107 | 77 | 0 | 100％ |
|  | 15 | 301 | 249 | 0 | 100％ |
|  | 16 | 257 | 206 | 0 | 100％ |
|  |  |  |  |  |  |
| EF3 | A | 485 | 361 | 17 | 96% |
|  | B | 192 | 148 | 4 | 97％ |
|  | C | 253 | 211 | 0 | 100％ |
|  | D | 131 | 68 | 0 | 100％ |
|  | E | 477 | 432 | 0 | 100％ |
|  | F | 454 | 271 | 136 | 66％ |
|  | G | 263 | 169 | 0 | 100％ |

**Table S2.** Oligonucleotide primers used in this study

| **Primer name** | **Primer sequence（5’-3’）** |
| --- | --- |
| pBacRNA-F | CCTAATACGACTCACTATAGGGAGACTTATTATATATATATTTTCTTGTT |
| pBacRNA-R | ATCGGTCTGTATATCGAGGTTTATTTATTAATTTGAA TAGATATT |
| Lsbnk-Prom-F | TTACGCGGCCGCTTTAGCTAGTTAGTTAGTTAGTTAGTT |
| Lsbnk-Prom-R | TTACCCATGGTTTTATTTCACTGCTCGTTTTGATTAC |
| Lshid-5UTR-F | GAGTTGATTTCGAACTACGAAGCGAGTAAG |
| Lshid-R | ATACACCGCCTCCCACACCC |
| SV40-BgIII-F | TAGTAAGCTTGGATAGATCTCTTTGTGAAGGAACCTTACT |
| SV40-XhoI-R | CCCCCTCGAGACATGATAAGATACATTGATGA |
| NcoIATGGCGNWStra | GCAGCCATGGCGGTAATTTTCTTTACGTATATCAAGTGTTACG |
| NWStra-SpeI | TGCATACTAGTCTCGTATTGACCGTGCCGTCCCTAATA |
| NSWtra-StuI | TTTTAGGCCTCTAATTTTTTGAGCAACATT |
| Lshidaa2-NSWtra | TCTGGCAAATAAAAGGGCACAGCCTAAACATAGAAAAGAATAATAAATTTATCATACA |
| NWStra-Lshidaa2 | TGTATGATAAATTTATTATTCTTTTCTATGTTTAGGCTGTGCCCTTTTATTTGCCAGA |
| LshidTGA-BgIII | TCTGAGATCTTCATCGGGCTACTAGAAATCCACAA |
| Lshidaa3-NSWtra | TCTGGCAAATAAAAGGGCACCTAAACATAGAAAAGAATAATAAATTTATCATACA |
| NWStra-Lshidaa3 | TGTATGATAAATTTATTATTCTTTTCTATGTTTAGGTGCCCTTTTATTTGCCAGA |
| tTAo-qRT-F | TGTTGAATGAAGTGGGTATTGAAGGATTGACTACTCG |
| tTAo-qRT-R | CCAAAGGGCAAAAGTGGGTGTGATGTCTATC |
| Lcbnk-qRT-F | AAACTTACTTCGCCTCGCTCCAACTC |
| Lcbnk-qRT-R | GCTGCTTGTGAAAACTGTATGCTGGG |
| LcGST1-F | GCCAGTGTCAGCACCTTTG |
| LcGST1-R | GCAACCTTCCCAGTTTTCATC |
| Lctra-F | CACAACAACTGCTTATCATCGGCAACAACAACTAC |
| LctraR-qRT | TTATTAGTTTTACAGCCCGTATTGCG |
